# Supplementary material for: Gold nanoclusters-assisted delivery of NGF siRNA for effective treatment of pancreatic cancer
Source: Nat Commun. 2017 Apr 25;8:15130. doi: 10.1038/ncomms15130 (PMC5414062; doi:10.1038/ncomms15130)
Supplement: Supplementary Information — Supplementary Figures, Supplementary Tables, Supplementary Methods and Supplementary References [file ncomms15130-s1.pdf]

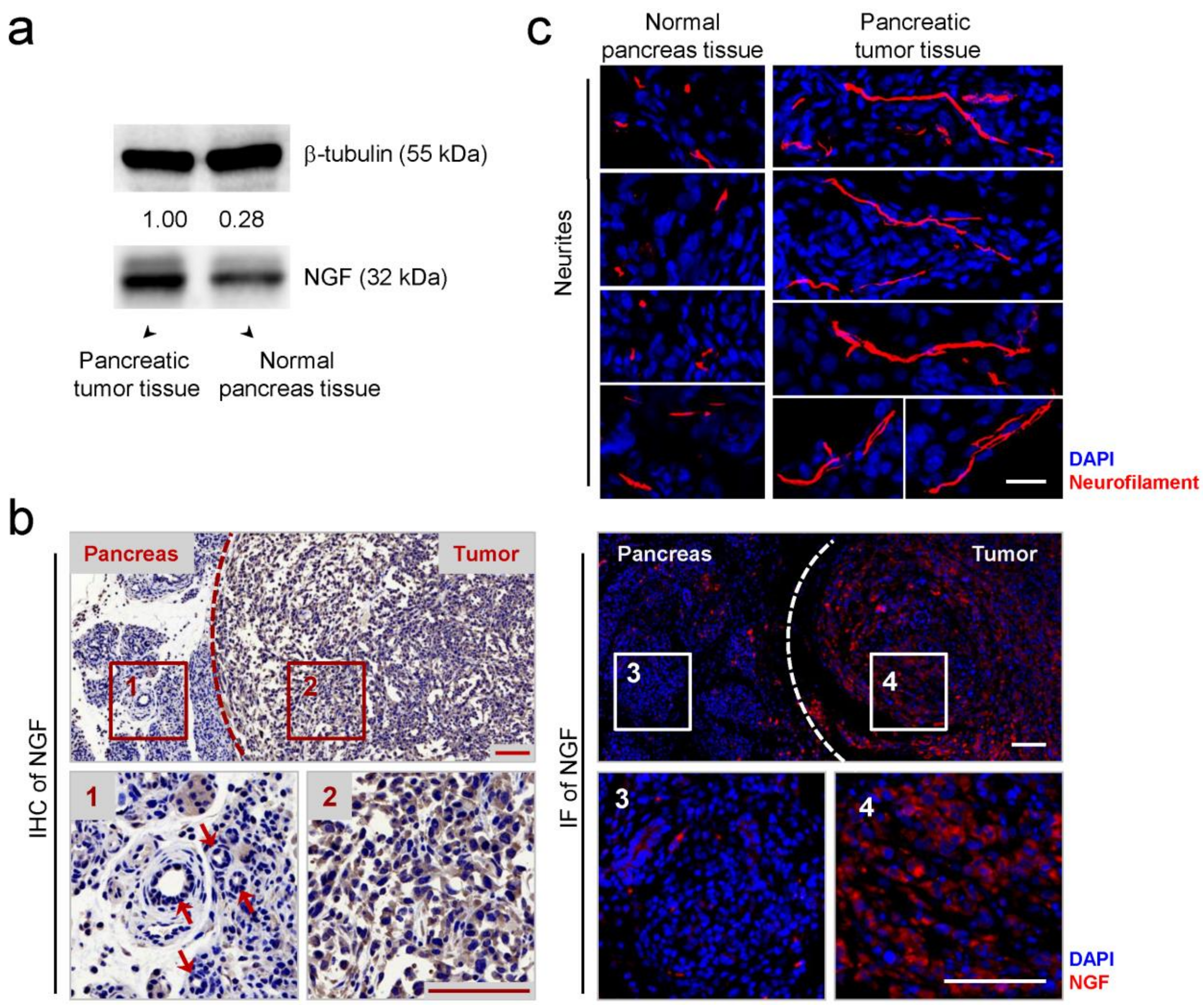

Supplementary Figure 1 | Expression of NGF protein and distribution of neurites in human pancreatic tumors. (**a**) Western blot detection of NGF protein in lysates of pancreatic tumor biopsies and normal pancreas tissue biopsies. NGF protein was detected at 32 kDa. We defined the optical density of NGF bands in pancreatic tumors being 1.0, the optical density of NGF in normal pancreas tissues was calculated to be 0.28 ± 0.08 (mean ± s.d. (n = 4); $p <$ 0.01; Student's *t*-test). (**b**) Detection of NGF in human pancreatic tumor biopsies by immunohistochemistry (IHC) and immunofluorescence (IF) to NGF antibody. The arrows in the IHC images represent the acinar structure of pancreas. Strong anti-NGF immunoreactivity was observed in pancreatic tumors. Scale bars: 100 µm. (**c**) Immunofluorescence staining of neurites in pancreatic tumor biopsy with neurofilament antibody, and the neural structure was reconstructed by Imaris 7.2 software. Scale bar: 20 µm. Neurites were shown in red, cell nuclei were counterstained with DAPI (blue).

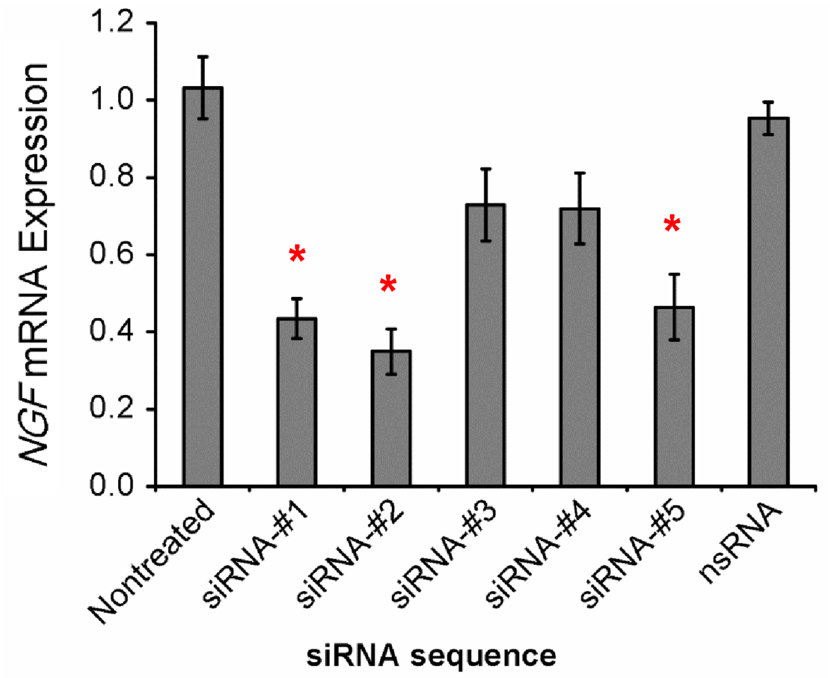

Supplementary Figure 2 | Screening of *NGF* siRNA sequences with RT-PCR. Five *NGF* siRNA sequences and one nonsense siRNA (nsRNA) were screened for *NGF* knockdown in Panc-1 cells using commercially available Lipofectamine® 2000 transfection agent. Each sequence was tested in triplicate over three independent experiments. Sequence siRNA-#2 resulted in the greatest knockdown of *NGF* mRNA compared with untreated control and nonsense siRNA (nsRNA). Mean ± s.d. (n = 3). * $p < 0.01$ compared with the nontreated control; Student's *t*-test.

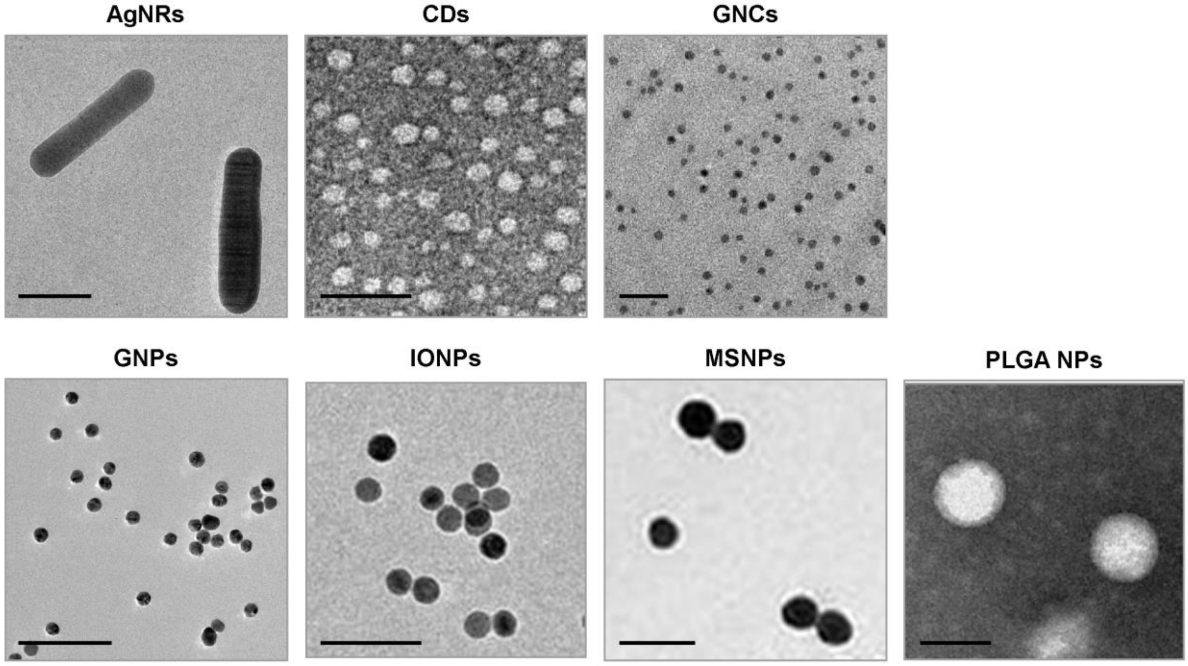

Supplementary Figure 3 | TEM images of nanomaterials screened for siRNA delivery in this study. AgNRs, silver nanorods. CDs, carbon dots. GNCs, gold nanoclusters. GNPs, gold nanoparticles. IONPs, iron oxide nanoparticles. MSNPs, mesoporous silica nanoparticles. PLGA NPs, poly(lactic-co-glycolic acid) nanoparticles. Scale bar is 20 nm for GNCs and 100 nm for the others.

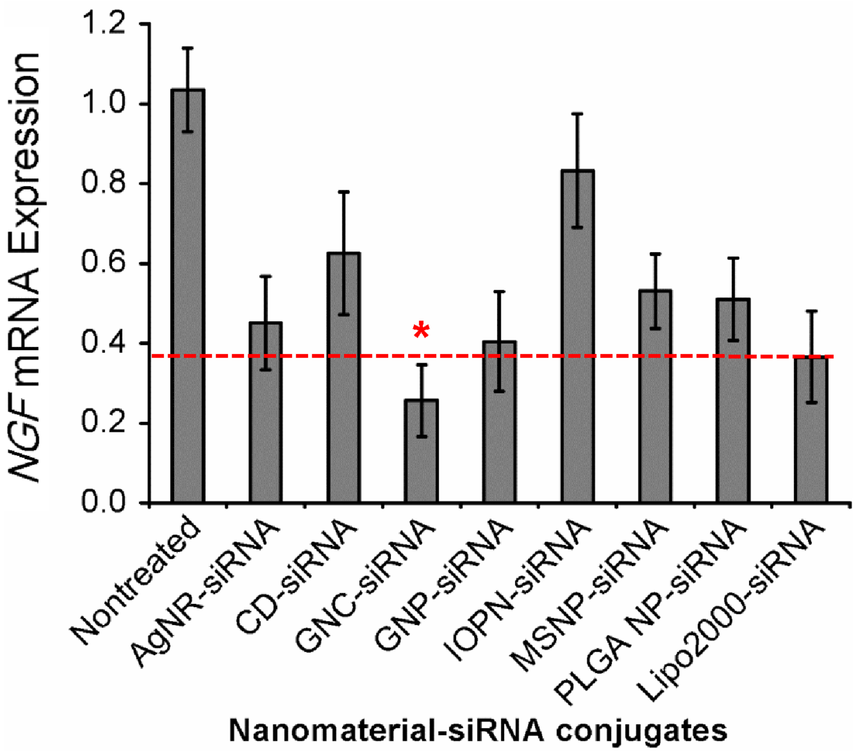

Supplementary Figure 4 | Screening of nanomaterials-siRNA conjugates for the greatest knockdown of *NGF*. Different nanomaterials-siRNA conjugates were prepared as described in *SI* text. Each conjugate (100 nM siRNA equivalent for each) was incubated with Panc-1 cells to determine the knockdown level of *NGF* mRNA by RT-PCR. Relative to the nontreated controls, GNC-siRNA complex exhibited the greatest knockdown of *NGF* mRNA level. The dotted line referred to the expression level of *NGF* mRNA in Panc-1 cells transfected with commercial available Lipofectamine® 2000 transfection agent (Lipo2000-siRNA, 100 nM siRNA equivalent) which served as a positive control. Mean ± s.d. (n = 3). * $p < 0.01$ compared with the nontreated control; Student's *t*-test.

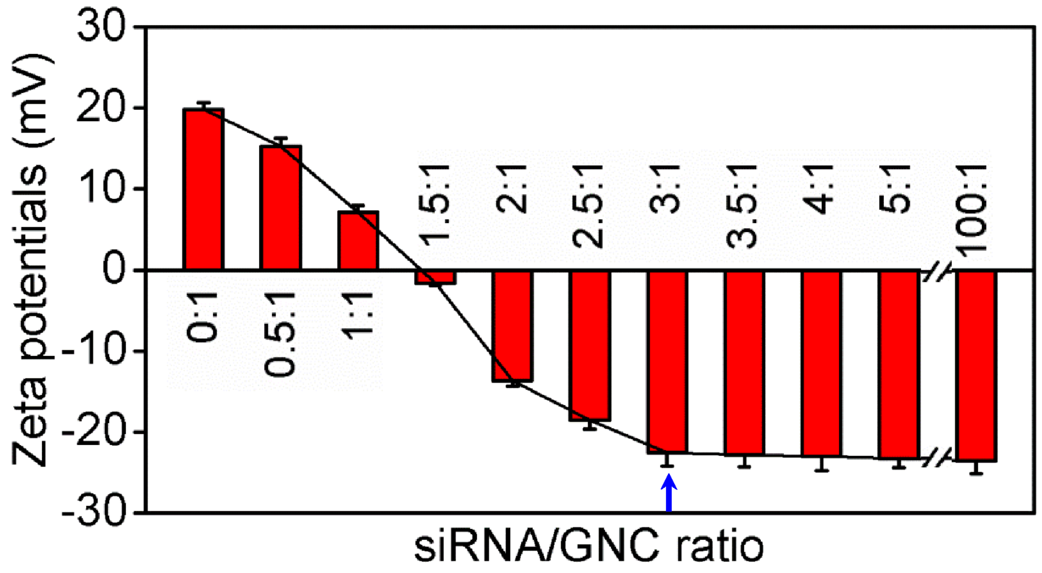

Supplementary Figure 5 | Characteristics of GNC-siRNA complex. Zeta potentials vs. siRNA/GNC ratio. Increasing amounts of siRNA were mixed with 1 µg ml^{-1} GNC solution. Mean ± s.d. (n = 6).

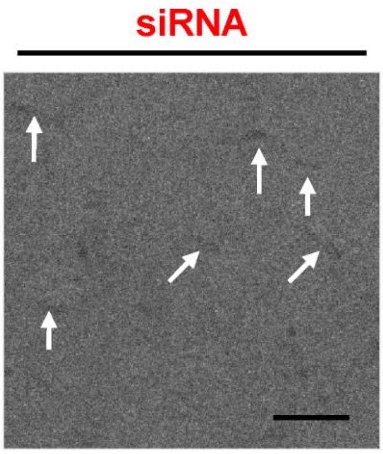

Supplementary Figure 6 | CryoTEM structure of siRNA duplex. The length and width of siRNA were measured at 7.3 ± 1.2 nm and 1.6 ± 0.3 nm, respectively, which are consistent with the size of siRNA in theory (7.5 nm in length and 2 nm in width). Scale bar: 10 nm.

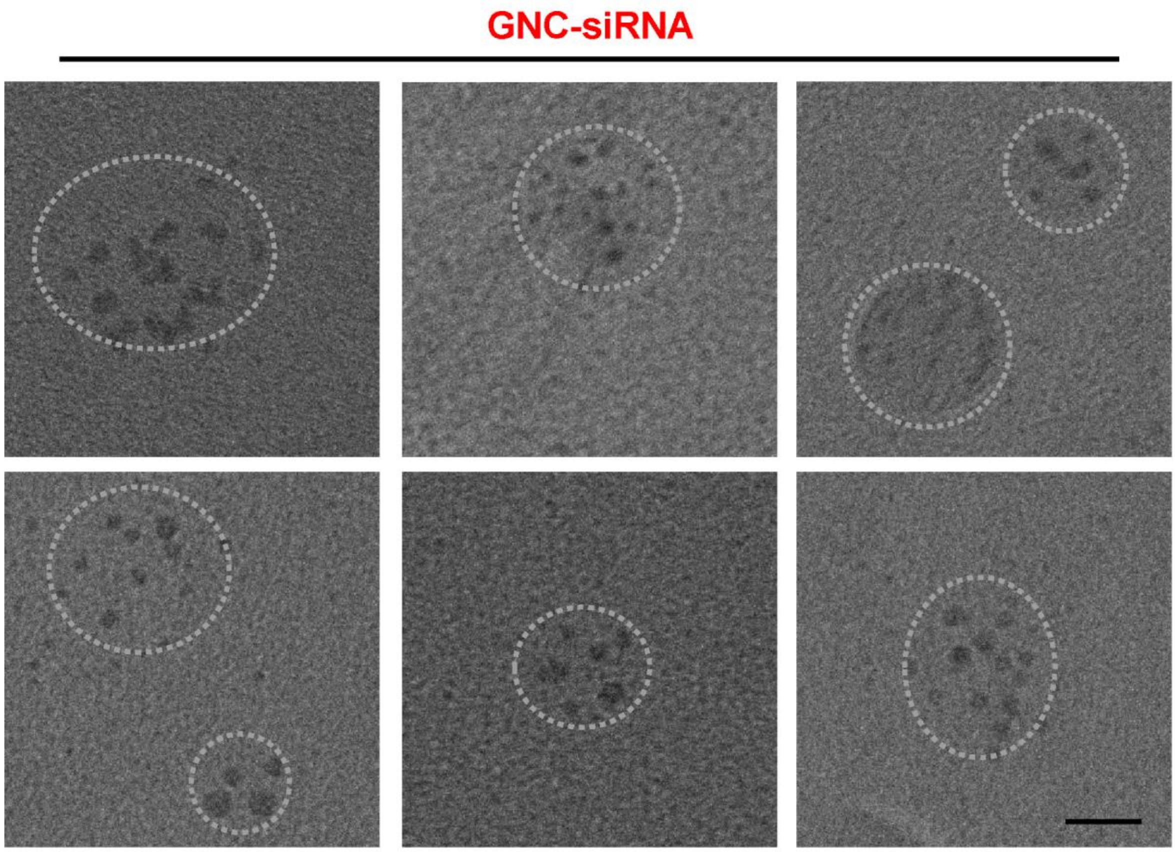

Supplementary Figure 7 | CryoTEM structure of GNC-siRNA complex. GNC-siRNA complex contained between 4 and 16 GNCs in the structure from the cryoTEM images. Scale bar: 10 nm.

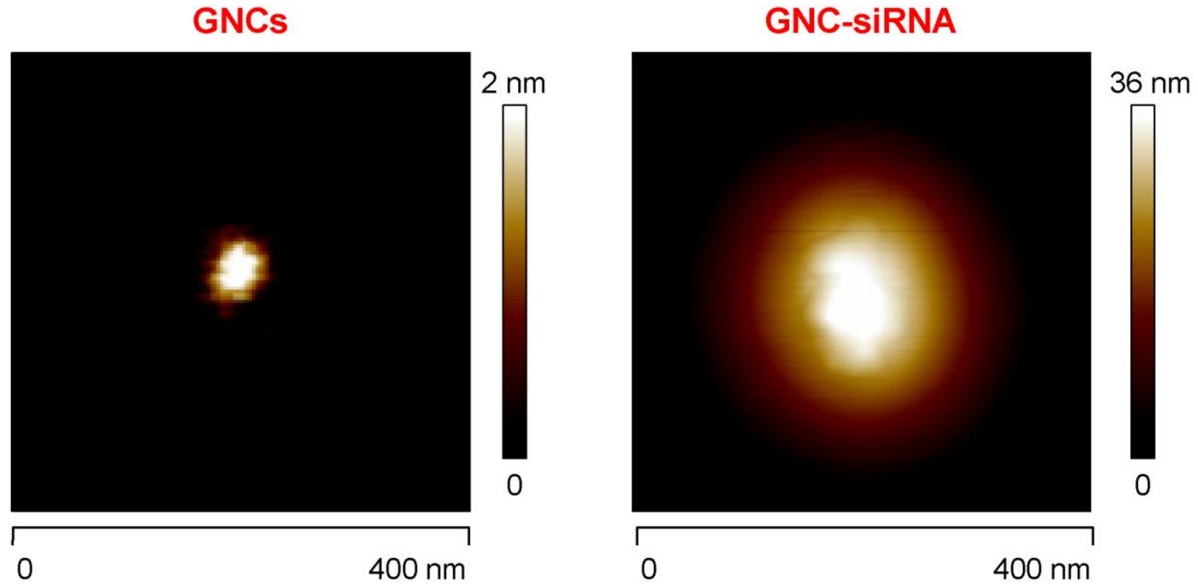

Supplementary Figure 8 | AFM structure of GNCs and GNC-siRNA complex.

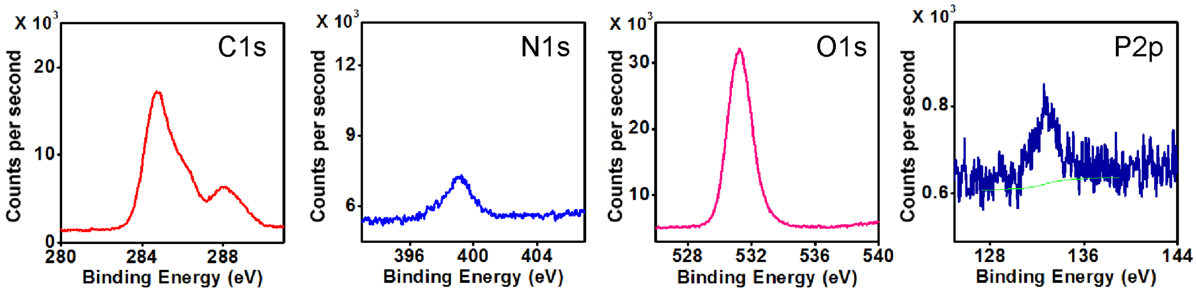

Supplementary Figure 9 | XPS analysis of *NGF* siRNA. XPS spectra for the chemical element of carbon (C), nitrogen (N), oxygen (O) and phosphor (P) of siRNA.

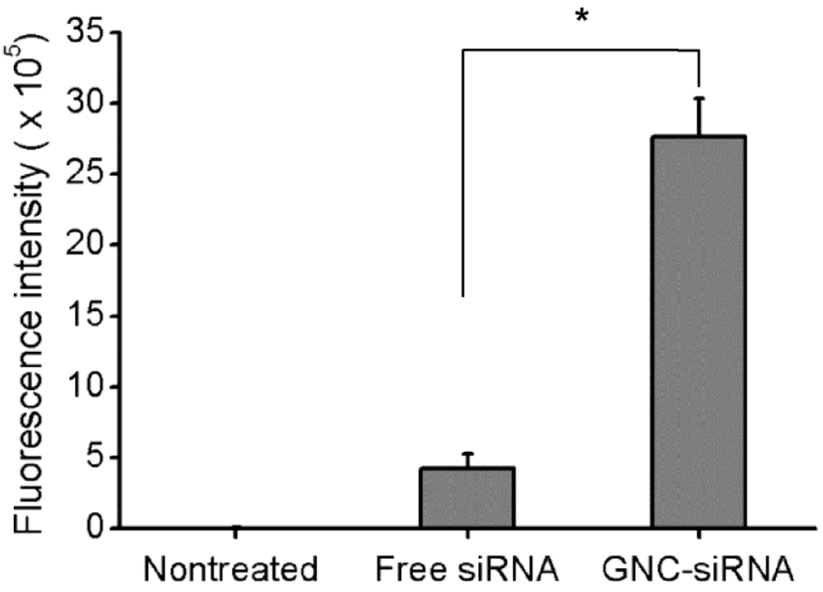

Supplementary Figure 10 | Quantification of cellular uptake of *NGF* siRNA in Panc-1 cells. *NGF* siRNA was labeled with Cy5 dye (Cy5-siRNA), and Panc-1 cells were incubated with different Cy5-siRNA formulations for 1 h. The fluorescence intensity of Cy5-siRNA in cells was quantified by ImageJ software (Mean ± s.d.; n= 20). * $p < 0.01$ compared with free siRNA; Student's *t*-test.

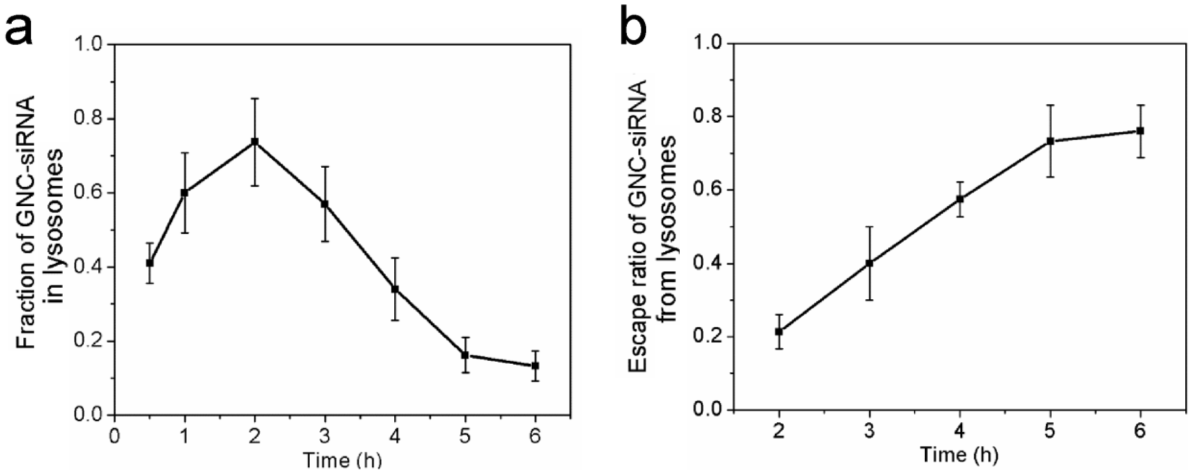

Supplementary Figure 11 | Intracellular trafficking and lysosomal escape of GNC-siRNA. The intracellular distribution of GNC-Cy5-siRNA (labeled in red) and lysosomes (labeled in green) was analyzed by ImageJ software. (**a**) Quantification of colocalization of GNC-siRNA in lysosomes over incubation time. (**b**) The escape ratio of GNC-siRNA from lysosomes at various time points. Mean ± s.d. (n = 6).

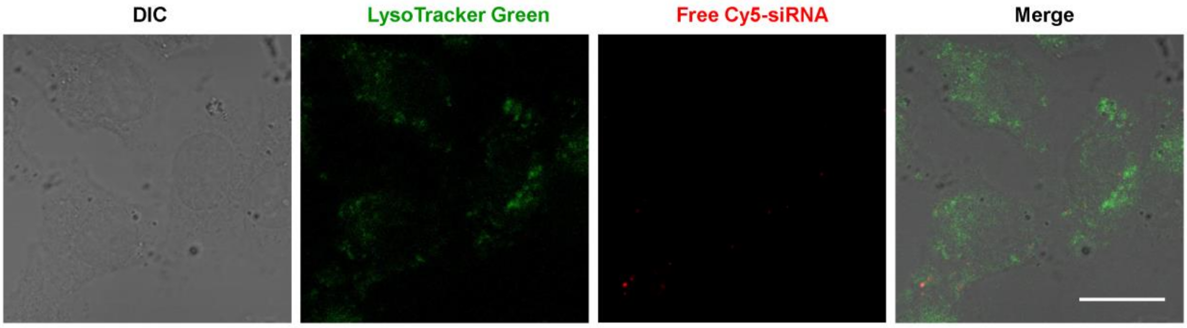

Supplementary Figure 12 | Colocalization of free siRNA and lysosomes in Panc-1 cells.. The lysosomes were stained with LysoTracker Green, and the Panc-1 cells were treated with free Cy5-siRNA (red) for 1 h. The cells were fixed and observed by confocal microscope. Scale bar: 20 µm.

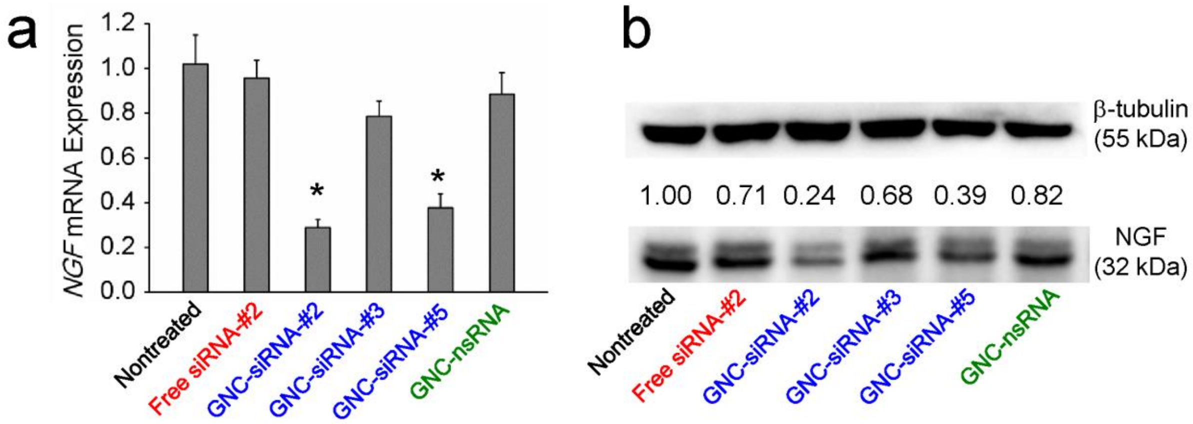

Supplementary Figure 13 | *NGF* gene silencing in Panc-1 cells with various *NGF* siRNA sequences. (**a**) Expression level of *NGF* mRNA in Panc-1 cells. (**b**) Expression level of NGF protein in Panc-1 cells. GNCs binding with *NGF* siRNA-#2, *NGF* siRNA-#3, *NGF* siRNA-#5 and nonsense siRNA (nsRNA) were named as GNC-siRNA-#2, GNC-siRNA-#3, GNC-siRNA-#5 and GNC-nsRNA, respectively. Mean ± s.d. (n = 4). * $p < 0.01$ compared with the nontreated control; Student's *t*-test.

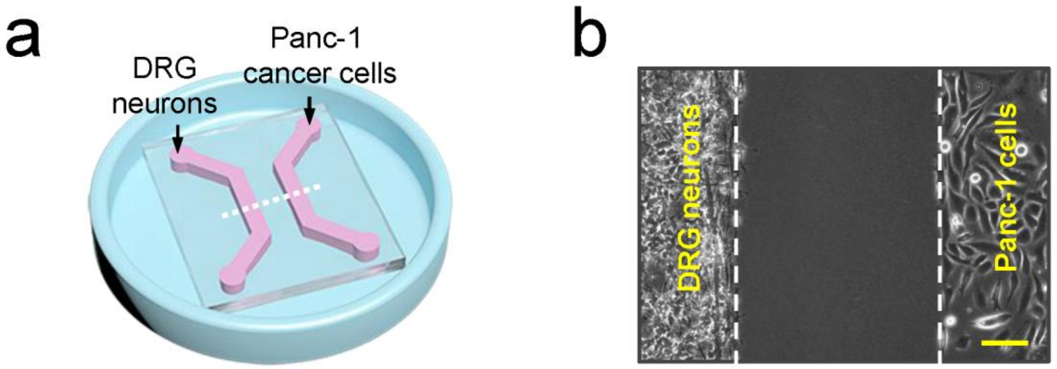

Supplementary Figure 14 | Co-culture of DRG neurons and Panc-1 cancer cells. (**a**) Scheme of microfluidic chip for co-culture of cells. (**b**) Phase-contrast images of co-culture of DRG neurons and Panc-1 cells after removal of the cover on the microfluidic chip. The neurite sprouting from DRG neurons was then evaluated. Scale bar: 100 µm.

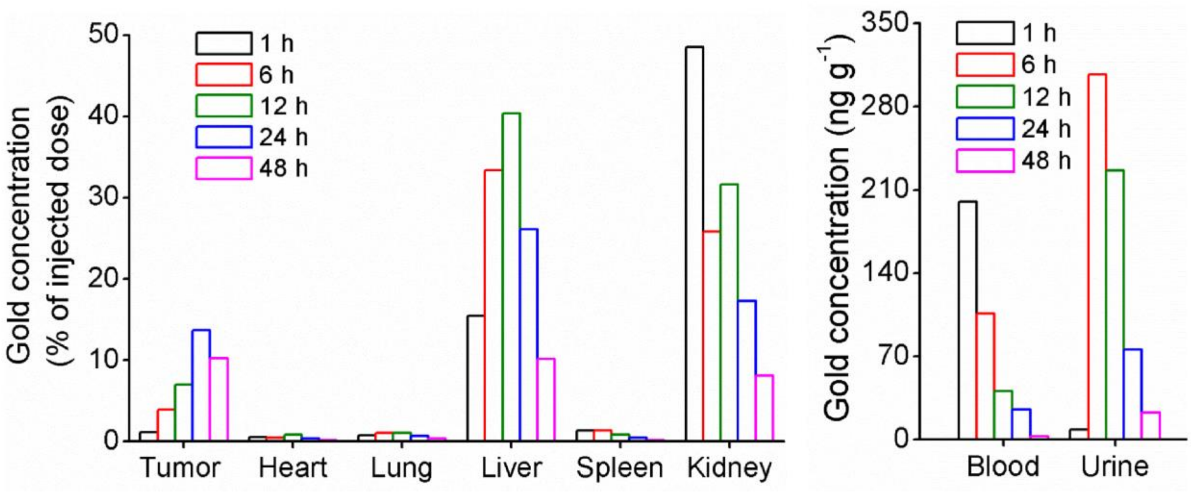

Supplementary Figure 15 | Biodistribution of gold in major organs, tumors, blood and urine of the mice. GNC-siRNA was injected into the mice via tail vein. At 1 h, 6 h, 12 h, 24 h and 48 h after injection, the gold concentration in major organs and tumors (expressed as % of injected dose), blood and urine (expressed as ng g^{-1}) of the mice was measured by ICP-MS.

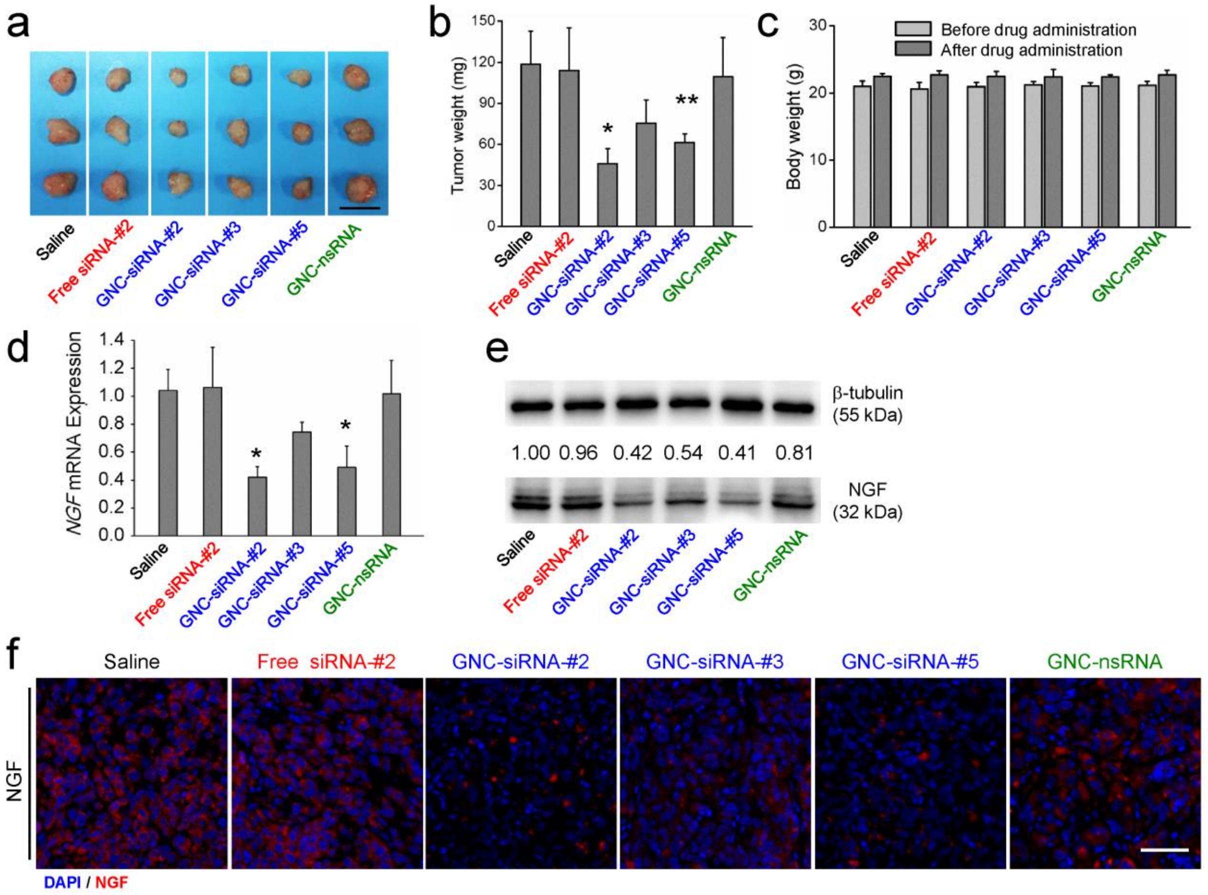

Supplementary Figure 16 | The anti-tumor and gene knockdown effects of various *NGF* siRNA sequences in orthotopic tumor model. Panc-1 cells were injected into the pancreas head of Balb/c nude mice, 15 days post implantation, the mice received intravenous injections of various formulations every 2 days for 6 injections. (**a**) *Ex vivo* tumor image and (**b**) Tumor weight at the end of the experiment. Scale bar: 1 cm. (**c**) Changes of mouse body weight before and after drug administration. (**d**) Expression level of *NGF* mRNA and (**e**) NGF protein in the orthotopic tumors. (**f**) Immunofluorescence staining of NGF (red) in tumor tissues, the cell nuclei were counterstained with DAPI (blue). Scale bar: 50 μm. GNCs binding with *NGF* siRNA-#2, *NGF* siRNA-#3, *NGF* siRNA-#5 and nonsense siRNA (nsRNA) were labeled as GNC-siRNA-#2, GNC-siRNA-#3, GNC-siRNA-#5 and GNC-nsRNA, respectively. Mean ± s.d. (n = 5 per group). Significant difference was from the saline control, * $p < 0.01$, ** $0.01 < p < 0.05$; Student's *t*-test.

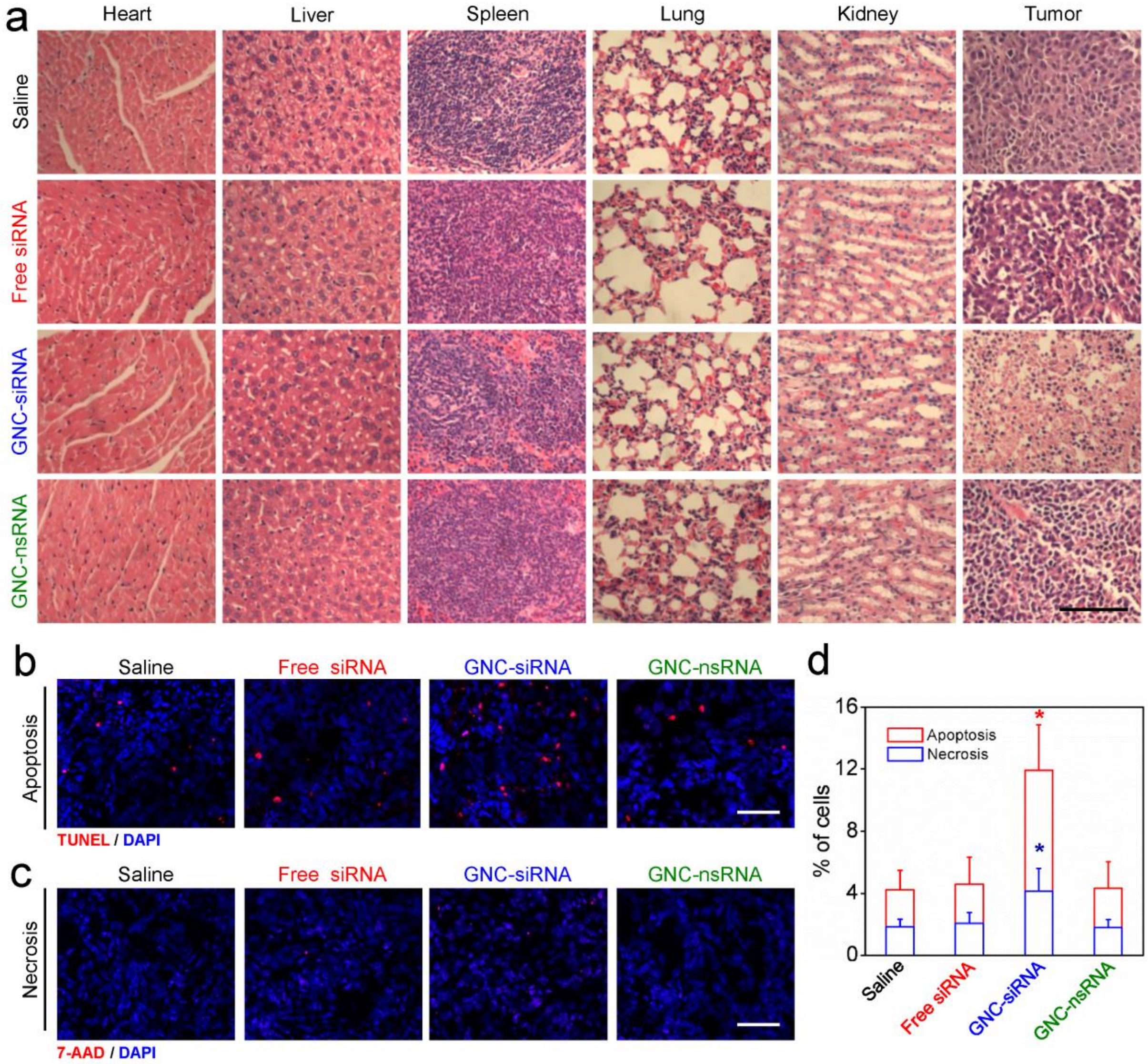

Supplementary Figure 17 | *In vivo* toxicity of GNC-siRNA complex in orthotopic tumor model. (**a**) H&E staining images of major organs and tumor tissues which were isolated 24 h after the last i.v injections of various drugs. Scale bar: 100 μm. (**b**) Detection of apoptosis in tumor tissues by TUNEL staining. TUNEL-positive cells were shown in red. Cell nuclei were shown by DAPI in blue. (**c**) Detection of necrosis in tumor tissues by 7-aminoactinomycin D (7-AAD) staining. Necrotic cells were shown in red, and cell nuclei were shown in blue. Scale bar: 50 μm. (**d**) Percentage of tumor cells positive to apoptosis or necrosis markers in the tumor tissues. The quantification was evaluated from at least 15 fields for each group (mean ± s.d.); * p < 0.01compated with saline control; Student's t-test.

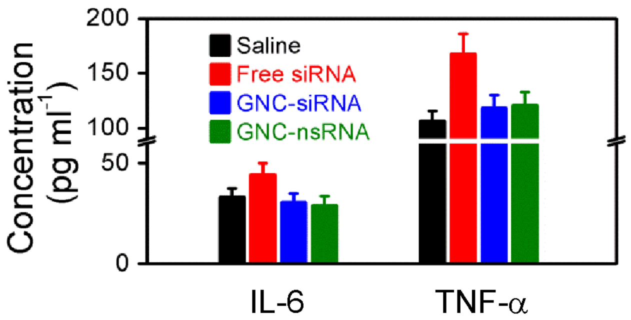

Supplementary Figure 18 | Imunnotoxicity analysis of blood of the mice at 24 h after drug injection. Interleukin-6 (IL-6) and tumor necrosis factor-α (TNF-α) were measurement by ELISA. Mean ± s.d. (n = 4).

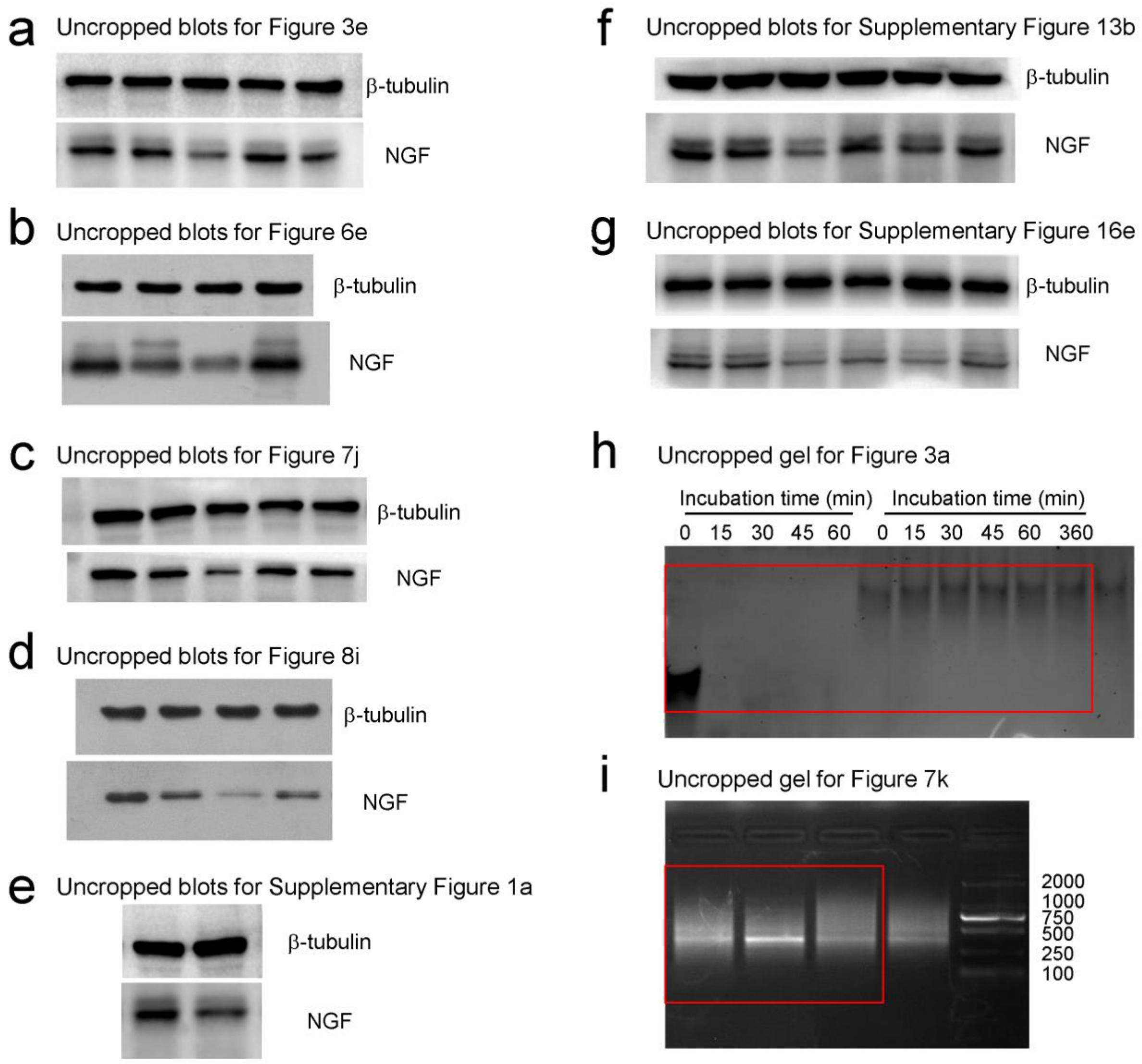

Supplementary Figure 19 | Uncropped western blots and gels. Western blots (**a**) for Figure 3e, (**b**) for Figure 6e, (**c**) for Figure 7j, (**d**) for Figure 8i, (**e**) for Supplementary Figure 1a, (**f**) for Supplementary Figure 13b, and (**g**) for Supplementary Figure 16e. (**h**) Polyacrylamide gel electrophoresis for Figure 3a. (**i**) 5'RACE assay on agarose gel for Figure 7k.

Supplementary Table 1 | Sequences of *NGF* siRNA

Name	Sequence
siRNA 1 sense	5’-CCAUGUUGUUCUACACUCU dTdT-3’
siRNA 1 antisense	3’-dTdT GGUACAACAAGAUGUGAGA-5’
siRNA 2 sense	5’-CCACAGACAUCAAGGGCAA dTdT-3’
siRNA 2 antisense	3’-dTdT GGUGUCUGUAGUUCCCGUU-5’
siRNA 3 sense	5’-GCACUGGAACUCAUAUUGU dTdT-3’
siRNA 3 antisense	3’-dTdT CGUGACCUUGAGUAUAACA-5’
siRNA 4 sense	5'-GACCACCGCCACAGACAUCdTdT-3’
siRNA 4 antisense	3’-dTdTCUGGUGGCGGUGUCUGUAG-5’
siRNA 5 sense	5'-GGGCAAGGAGGUGAUGGUGdTdT-3’
siRNA 5 antisense	3’-dTdTCCCGUUCCUCCACUACCAC-5’
nsRNA sense	5’-GCUGACCCUGAAGUUCAUCdTdT-3’
nsRNA antisense	3’-dTdTCGACUGGGACUUCAAGUAG-5’

siRNA sequences were determined from the National Center for Biotechnology Information database of *NGF* (accession number NM_002506).

Supplementary Table 2 | Sequences of primers used for RT-PCR

Name	Sequence
NGF forward	5’-CAACAGGACTCACAGGAGCA-3’
NGF reverse	5’-ACCTCTCCCAACACCATCAC-3’
RPLP0 forward	5’- GCAGGTGTTTGACAACGGCAG-3’
RPLP0 reverse	5’- GATGATGGAGTGTGGCACCGA-3’

Supplementary Table 3 | Characterization of nanomaterials-siRNA conjugates

	Size by TEM (nm)	Loading capacity of siRNA (original data)	Loading capacity of siRNA (converted data)
AgNRs	L: 220 ± 10.6<br>W: 50 ± 2.7	266 mg siRNA per g AgNRs	20 μmol siRNA per g AgNRs
CDs	21.5 ± 3.3	65 mg siRNA per g CDs	4.88 μmol siRNA per g CDs
GNCs	2.6 ± 0.5	3000 mg siRNA per g GNCs	226 μmol siRNA per g GNCs
GNPs	13.0 ± 1.1	34.79 mg siRNA per g GNPs	2.62 μmol siRNA per g GNPs
IOPNs	30 ± 2.5 nm	26.6 mg siRNA per g IOPNs	2 μmol siRNA per g IOPN
MSNPs	50 ± 5.6 nm	133 mg siRNA per g MSNPs	10 μmol siRNA per g MSNPs
PLGA NPs	109 ± 11 nm	5.32 mg siRNA per g PLGA	0.4 μmol siRNA per g PLGA NPs

Supplementary Table 4 | Characteristics of GNCs and GNC-siRNA complex by cryoTEM and DLS

	Size by cryoTEM (nm)	Hydrodynamic size by DLS (nm)	Polydispersity index (PDI)	Zeta potential (mV)
GNCs	2.6 ± 0.5	6.6 ± 0.8	0.111 ± 0.008	19.9 ± 0.8
GNC-siRNA	16.6 ± 3.0	70.2 ± 8.1	0.224 ± 0.016	-22.6 ± 1.6

Supplementary Table 5 | Relative atomic composition (%) in samples by XPS

	Au (%)	S (%)	C (%)	N (%)	O (%)	P(%)
siRNA	ND	ND	59.84	4.03	35.18	0.94
GNCs	1.34	4.46	59.85	4.78	29.57	ND
GNC-siRNA	0.20	2.41	60.58	6.04	30.23	0.54

ND, not detected

Supplementary Table 6 | Biochemical parameters of blood from the mice at 24 h after last injection of various drugs

	ALT ($U\ l^{-1}$)	AST ($U\ l^{-1}$)	BUN (mM)	CREA (μM)
Saline	48 ± 11	132 ± 9	5.6 ± 0.6	32 ± 4
Free siRNA	154 ± 32	193 ± 37	7.2 ± 0.9	24 ± 3
GNC-siRNA	51 ± 12	168 ± 21	8.0 ± 0.8	41 ± 4
GNC-nsRNA	76 ± 21	160 ± 15	6.4 ± 0.9	25 ± 3
Reference	17-132	54-298	2.8-11.7	18-80

ALT, alanine amino transferase; AST, aspartate amino transferase; BUN, blood urea nitrogen; CREA, creatinine.

Supplementary Methods

Preparation of nanomaterials-siRNA conjugates. We have prepared a broad diversity of nanomaterials conjugated with *NGF* siRNA for the delivery of *NGF* siRNA based on the nanomaterials available in our labs. The nanomaterials used for siRNA delivery in this study included silver nanorods (AgNRs), carbon dots (CDs), gold nanoclusters (GNCs), gold nanoparticles (GNPs), iron oxide nanoparticles (IONPs), mesoporous silica nanoparticle (MSNPs), and poly(lactic-co-glycolic acid) nanoparticles (PLGA NPs). The morphology of the nanomaterials was presented in Supplementary Fig. 3. All nanomaterials and stock solutions were prepared using nuclease-free water (Gibco).

AgNR-siRNA conjugates. Silver nanorods (AgNRs, 220 nm length, 50 nm width) were prepared as follows: 0.5 ml of 1 M $AgNO_3$ and 2.5 ml of 1 M PVP (MW 40 kDa) were added to continuous stirring 25 mL of 10 µM PEG_{600} at room temperature at 3 minutes interval. This mixture was stirred further 5 minutes before transferring into oil bath maintained at 70 °C. After 1 hour, the temperature of the bath was increased to 100 °C and maintained for 20 hours. The final AgNRs were precipitated in a large amount of ethanol and water.

The obtained AgNRs (65 µg ml^{-1}) were dropped into 3 mg ml^{-1} poly(styrene sulfonate) (PSS) solution and then 3 mg ml^{-1} poly(dimethyl- diallylammonium chloride) (PDDA) solution to switch the surface to positive charge (40 mV). The siRNA solution with a series of increased concentration was added into the AgNR solution (5 µg ml^{-1}) to determine the saturation concentration of siRNA by zeta-potential measurement. The solution was centrifuged at 4000 × g for 30 min and washed three times in water, the final product were labeled as AgNR-siRNA. In this work, the loading amount of siRNA on AgNRs was about 266 mg siRNA per g AgNRs, equivalent to 20 µmol siRNA per g AgNRs.

CD-siRNA conjugates. Amino-functionalized carbon dots (CDs, 21.5 ± 3.3 nm) were obtained by hydrothermal carbonization of chitosan. In brief, 5 mg ml^{-1} chitosan was dispersed in distilled water, followed by hydrothermal treatment at 180°C for 12 h. After cooling, the solution was centrifuged at 14000 × g for 15 min.

The amine-functionalized CDs were activated by N-(γ-maleimidobutyryloxy)

sulfosuccinimide ester to introduce maleimide groups on CDs. siRNA was modified with thiol group at the 3' end of the sense strand (SH-siRNA). After addition of SH-siRNA into the CD solution, the concentration of NaCl was brought up to 300 mM, which increased the coverage of siRNA on the surfaces of CDs. After 4 h, the mixture was shaken for ~24 h to complete the siRNA functionalization process. The particles were ultra-filtrated three times to remove the unbound siRNA and impurities, and the products were labeled as CD-siRNA. In this work, the loading amount of siRNA was about 65 mg siRNA per g CDs, equivalent to 4.88 µmol siRNA per g CDs.

GNC-siRNA conjugates. The positively charged gold nanoclusters (GNCs, 2.66 ± 0.45 nm) were synthesized from the reduction of Au^{3+} in the presence of glutathione and oligoarginine that carries amine-derived positive charge.[1] In brief, $HAuCl_4$ solution (100 mM), thiolate containing glutathione (GSH, 150 mM) and oligoarginine CRRRRRRRRR (CR_9, 75 mM) were mixed with ultrapure water at 25 °C. The reaction mixture was heated to 70 °C under gentle stirring (500 rpm) for 24 h. An aqueous solution of gold nanoclusters with light-green color was formed, and they could be stored at 4 °C for 6 months with negligible changes in their optical properties.

The positively charged GNCs (1 µg ml^{-1}) were mixed with siRNA solution in ultrapure water, and shaken on a bench top shaker for 1 h to complete the binding of siRNA onto the GNCs via electrostatic interaction. The *NGF* siRNA was added into the GNC solution in different concentrations to determine the saturated concentration of siRNA solution, with the weight ratio of siRNA to GNCs varied from 0:1 to 100:1. The final product was abbreviated as GNC-siRNA. In this work, the loading amount was about 3000 mg siRNA per g GNCs, equivalent to 226 µmol siRNA per g GNCs.

GNP-siRNA conjugates. GNPs (13.0 nm ± 1.1 nm) were prepared by the citrate-mediated reduction of $HAuCl_4$ in aqueous solution according to our previous reports.[2]

For binding siRNA onto GNPs, siRNA was modified with thiol group at the 3' end of sense strand (SH-siRNA). The SH-siRNA was added to the solution of GNPs (5 mM), sonicated, and shaken on a bench top shaker for 4 h. After, the NaCl concentration in the

solution was brought up to 350 mM to allow the assembly of SH-siRNA onto GNPs via thiol-Au assembly. After sonication, the solution was shaken overnight at room temperature to complete the functionalization process. The solution was centrifuged at 13000 × g at 4 °C for 30 min to remove the unbound siRNA for three times. The obtained particles were name as GNP-siRNA. In this work, the loading amount was about 34.79 mg siRNA per g GNPs, equivalent to 2.62 µmol siRNA per g GNPs.

IOPN-siRNA conjugates. Amine-functionalized iron oxide nanoparticles (IONPs, 30 nm ± 2.5 nm) were purchased from Ocean Nanotech, USA. The siRNA was coated on the positively charged IONPs via electrostatic interaction. The siRNA was added into the IONP solution (100 µg ml^{-1}) in a series of different concentrations to determine the saturated concentration of siRNA solution. The particles were centrifuged at 7000 × g for 30 min to remove the unbound siRNA for three times. The final product was labeled as IOPN-siRNA. In this work, the loading amount of siRNA was about 26.6 mg siRNA per g IOPNs, equivalent to 2 µmol siRNA per g IOPNs.

MSNP-siRNA. Mesoporous silica nanoparticles (MSNPs, 50 nm ± 5.6 nm) were purchased from Sigma-Aldrich. The MSNPs (10 mg ml^{-1}) were suspended in 10 ml 2.5 mg ml^{-1} polyethyleneimine (PEI, 10 kDa), and stirred at room temperature for 30 min. The positively charged particles (PEI-MSNPs) were collected by centrifugation at 5000 rpm and washed three times with purified water.

The siRNA was added and coated onto the PEI-MSNPs via electrostatic interaction. The siRNA was added into the solution of PEI-MSNPs in a series of different concentrations to determine the saturation concentration of siRNA solution. The particles were centrifuged at 5000 rpm in purified water for three times to remove the unbound siRNA. The obtained formulation was named as MSNP-siRNA. In this work, the loading amount of siRNA was about 133 mg siRNA per g MSNPs, with equivalent 10 µmol siRNA per g MSNPs.

PLGA NP-siRNA conjugate. PLGA nanoparticles (109 nm ± 11 nm) were prepared using nanoprecipitation. Briefly, 20 mg of PLGA-PEG (Sigma-Aldrich) was dissolved in 1 ml

methylene chloride, and added drop wise into water. The NPs were stirred for 1 h, and the remained organic solvent was removed by vacuum evaporation. The NPs were centrifuged at 5000 × g for 15 min and washed three times with ultrapure water.

The PLGA NPs (1 mg ml^{-1}) was stirred with 50 μg ml^{-1} PEI solution (10 kDa) to switch the surface to positive charge. And the siRNA solution with different concentrations was added into the PLGA solution, to determine the saturation concentration of siRNA. The particles were centrifuged at 5000 rpm in purified water for three times to remove the unbound siRNA. In this work, the loading amount of was about 5.32 mg siRNA per g PLGA, with equivalent 0.4 μmol siRNA per g PLGA NPs.

Characterization of nanocarrier-siRNA conjugates. The morphology of the nanomaterials was observed by transmission electron microscope (TEM). The zeta potential of the nanoparticles was characterized by dynamic light scattering (DLS, Malvern Instruments Ltd.). In the above preparations, the negatively charged siRNA was titrated into each solution to determined the saturation of siRNA binging. The saturation of siRNA was determined by measuring the zeta potential of the solution, till a stable and negatively charged solution was obtained.

To characterize the amount of siRNA onto the nanomaterials-siRNA conjugates, the Cy5 dye (excitation/emission at 649/670nm) labeled siRNA was used for quantification. The concentration of Cy5-siRNA in each solution was determined by measuring the fluorescence intensity of solution at 649 nm excitation and 670 nm emission on an EnSpire Multimode Plate Reader (PerkinElmer), and calculated with a calibration curve of a series of Cy5-siRNA dilution.

Detection of apoptosis. We detected the apoptosis in tumor tissue sections by TUNEL (terminal transferase dUTP nick end labeling) assay, since TUNEL is a common method for detecting DNA fragmentation that from apoptotic signaling cascade. The slices were fixed in 4% PFA, permeabilized with 0.3% Triton X-100, and incubated with a TUNEL reaction mixture (Roche, 12156792910) for 1 h in a dark, humidified chamber. Cell nuclei were counterstained with DAPI for 10 min to label all cells. The sections were mounted and

observed with confocal microscopy. Each image was examined at a magnification of 400×. Cells undergoing apoptosis (TUNEL-positive cells) were labeled red, and cell nuclei were labeled blue. The apoptosis level was calculated as the percentage of TUNEL-positive cells in the slices using ImageJ software.

Detection of necrosis. We assessed the necrosis in tumor tissue sections using a modified necrosis detection kit (Abcam, 176749). The necrosis was detected by 7-aminoactinomycin D (7-AAD, a dye labels the nucleus of damaged cells in necrosis, ex/em = 550/650 nm), the samples were incubated with 7-AAD solution at room temperature for 60 min, and cell nuclei were counterstained with DAPI for 10 min. Necrotic cells stained with 7-AAD were shown in red, and cell nuclei were labeled in blue. The necrosis level was calculated as the percentage of 7-AAD-positive cells in the slices.

Supplementary References

1 Xie, J., Zheng, Y. & Ying, J. Y. Protein-directed synthesis of highly fluorescent gold nanoclusters. *J. Am. Chem. Soc.* **131**, 888-889 (2009).

2 Xianyu, Y. L., Wang, Z. & Jiang, X. Y. A plasmonic nanosensor for immunoassay via enzyme-triggered click chemistry. *ACS Nano* **8**, 12741-12747 (2014).
